# A new role of the Rac-GAP β2-chimaerin in cell adhesion reveals opposite functions in breast cancer initiation and tumor progression

**DOI:** 10.18632/oncotarget.8597

**Published:** 2016-04-05

**Authors:** Victoria Casado-Medrano, Laura Barrio-Real, Ginesa García-Rostán, Matti Baumann, Oliver Rocks, María J. Caloca

**Affiliations:** ^1^ Instituto de Biología y Genética Molecular (IBGM), Consejo Superior de Investigaciones Científicas (CSIC), Universidad de Valladolid, 47003 Valladolid, Spain; ^2^ Current address: Department of Pharmacology, Perelman School of Medicine, University of Pennsylvania, Philadelphia, PA 19104, USA; ^3^ Max Delbrück Center for Molecular Medicine, 13125 Berlin, Germany

**Keywords:** β2-chimaerin, breast cancer, E-cadherin, metastasis, Rac1

## Abstract

β2-chimaerin is a Rac1-specific negative regulator and a candidate tumor suppressor in breast cancer but its precise function in mammary tumorigenesis *in vivo* is unknown. Here, we study for the first time the role of β2-chimaerin in breast cancer using a mouse model and describe an unforeseen role for this protein in epithelial cell-cell adhesion. We demonstrate that expression of β2-chimaerin in breast cancer epithelial cells reduces E-cadherin protein levels, thus loosening cell-cell contacts. *In vivo*, genetic ablation of β2-chimaerin in the MMTV-Neu/ErbB2 mice accelerates tumor onset, but delays tumor progression. Finally, analysis of clinical databases revealed an inverse correlation between β2-chimaerin and E-cadherin gene expressions in Her2+ breast tumors. Furthermore, breast cancer patients with low β2-chimaerin expression have reduced relapse free survival but develop metastasis at similar times. Overall, our data redefine the role of β2-chimaerin as tumor suppressor and provide the first *in vivo* evidence of a dual function in breast cancer, suppressing tumor initiation but favoring tumor progression.

## INTRODUCTION

Rac1 is a member of the Rho family of GTPases which regulates a variety of cellular processes including proliferation, survival, cell adhesion and motility [1]. Deregulation of such processes are hallmarks of cancer and not surprisingly, altered Rac1 activity has been associated with malignant transformation in various types of cancer including breast cancer [2–4]. Studies to determine the molecular mechanisms responsible for the hyperactivation of Rac1 in tumors unveiled that genetic alterations on this GTPase are very rare [5, 6]. Instead, deregulation of Rac1 typically occurs at the level of their regulatory proteins, the guanine nucleotide exchange factors (GEFs), the GDP dissociation inhibitors (GDIs), and the GTPase activating proteins (GAPs) [7–9].

β2-chimaerin is one of the few Rac-GAP proteins that have been implicated in breast cancer [7]. This protein is a product of the *CHN2* gene which also encodes the testis-specific β1-chimaerin and a recently identified β3-chimaerin isoform [10, 11]. β2-chimaerin possesses three conserved structural domains; an SH2 domain, a C1 domain that bind diacylglycerol (DAG), and a catalytic GAP domain with specificity for the Rac GTPase [12–14]. This combination of structural domains confers β2-chimaerin the property to regulate Rac activity via receptors coupled to DAG generation such as the epidermal growth factor (EGF) receptors [14, 15]. Deregulation of the *CHN2* gene has been associated with human pathologies including mental disorders [16, 17], insulin resistance [18], and cancers such as glioblastoma, hepatosplenic T-cell lymphoma and breast cancer [19–21].

The evidences that link β2-chimaerin and breast cancer are very limited but suggest a tumor suppressor role for this protein. For example, downregulation of β2-chimaerin has been reported in human breast cancer cell lines and in a few number of human breast cancer samples [20]. Conversely, restoration of β2-chimaerin in breast cancer cells inhibits proliferation, impairs migration and reduces the tumorigenic potential [20, 22–24]. The molecular mechanisms underlying these anti-tumorigenic effects of β2-chimaerin are only partially elucidated. The inhibition of Rac1 by β2-chimaerin reduces cyclin D1 levels and pRb phosphorylation, thus impairing G1/S cell cycle progression [20]. Furthermore, inhibition of cell proliferation by β2-chimaerin is also observed in response to heregulin stimulation, ligands for the ErbB receptors that play important roles in breast tumorigenesis [24, 25]. How β2-chimaerin affects breast cancer cell migration and invasion is less studied, although it seems clear that its Rac1-specific GAP activity has a role in these processes by modulating actin dynamics [15, 22]. Interestingly, the ablation of the *Drosophila* orthologue of chimaerin produces aberrant cell contacts in the eye epithelium, suggesting a role for this protein in the regulation of cell-cell adhesion [26].

The above studies suggest that β2-chimaerin can regulate various processes important in breast cancer development and progression, but the data so far are too limited to validate this protein as a target of therapeutic interest. In this study we have used a combination of *in vitro* analysis in breast cancer cell lines, a well-defined mouse model of breast cancer, and bioinformatics analyses of human breast cancer databases to delineate the role of β2-chimaerin in breast cancer.

## RESULTS

### β2-chimaerin alters E-cadherin expression in MCF7 breast cancer cells

**Figure 1:**
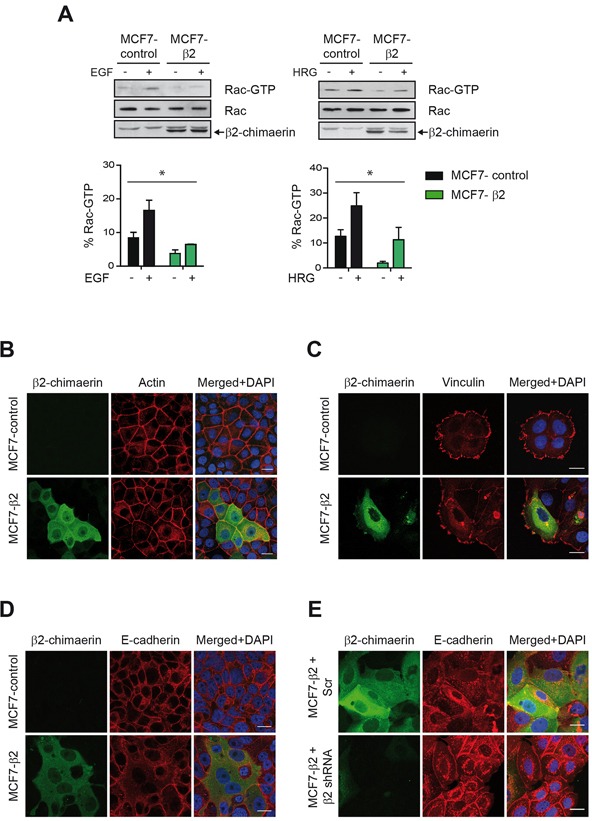
The Rac-GAP β2-chimaerin alters E-cadherin in MCF7 cells **A.** Active Rac1 in MCF7-control and MCF7-β2 cells after stimulation with EGF (100 ng/ml) or HRG (10 ng/ml). Densitometric analysis of Rac1-GTP levels normalized to the total Rac1 in two different experiments is shown in the histograms. Bars are the mean ± s.e.m. (*P < 0.05, ANOVA test). **B.** Immunofluorescence images of actin staining in MCF7-control and MCF7-β2 cells in confluent cultures. Note that cells expressing β2-chimaerin-EGFP have a bigger size. **C.** Immunostaining of vinculin in MCF7-control and MCF7-β2 cells grown on collagen IV-coated glasses. **D.** Immunostaining of E-cadherin in MCF7-control and MCF7-β2 cells. **E.** Immunostaining of E-cadherin in β2-chimaerin-silenced MCF7-β2 cells In **B-E**, panels show representative confocal immunofluorescence images from one of three independent experiments. Scale bars: 20 μm.

β2-chimaerin has been proposed to influence cytoskeleton-mediated processes in different cell types by inhibiting Rac activity [15, 22, 27, 28], but these functions are only poorly characterized in breast cancer epithelial cells. To further investigate this issue, we analyzed the effect of the ectopic expression of β2-chimaerin in MCF7 cells, a breast cancer epithelial cell line with undetectable levels of endogenous β2-chimaerin [20]. To this end, we generated a MCF7 cell line stably expressing EGFP-tagged β2-chimaerin. Quantitative real-time RT-PCR analysis revealed that these cells had ~10-fold increase over the endogenous β2-chimaerin mRNA levels in normal human breast tissue (Supplementary Figure S1). We corroborated that the expressed β2-chimaerin-EFGP was fully functional as determined by its ability to inhibit Rac activation by EGF or heregulin (HRG) as previously described [20, 24] (Figure 1A). Since Rac1 activity is essential for the control of the actin cytoskeleton, cell-matrix adhesion and cell-cell adhesion, we examined the effects of β2-chimaerin expression on these processes by staining the actin cytoskeleton as well as vinculin and E-cadherin, main components of focal adhesions and adherens junctions (AJs) respectively. Confocal immunofluorescence analysis revealed that cells expressing β2-chimaerin (MCF7-β2) retained the cobblestone morphology typical of epithelial cells and displayed the characteristic circumferential band of F-actin encircling the cells at the perijunctional level (Figure 1B). Notably, MCF7-β2 cells exhibited in average a larger cell size than control cells. The formation of focal adhesions, as measured by the appearance of vinculin clusters, was not affected by the expression of β2-chimaerin (Figure 1C). However, the E-cadherin signal at cell-cell contact was strongly reduced (Figure 1D). This effect is directly associated with the expression of β2-chimaerin, since silencing of this protein in the MCF7-β2 cells with a specific shRNA restored the normal levels of E-cadherin at the cell junctions (Figure 1E).

**Figure 2:**
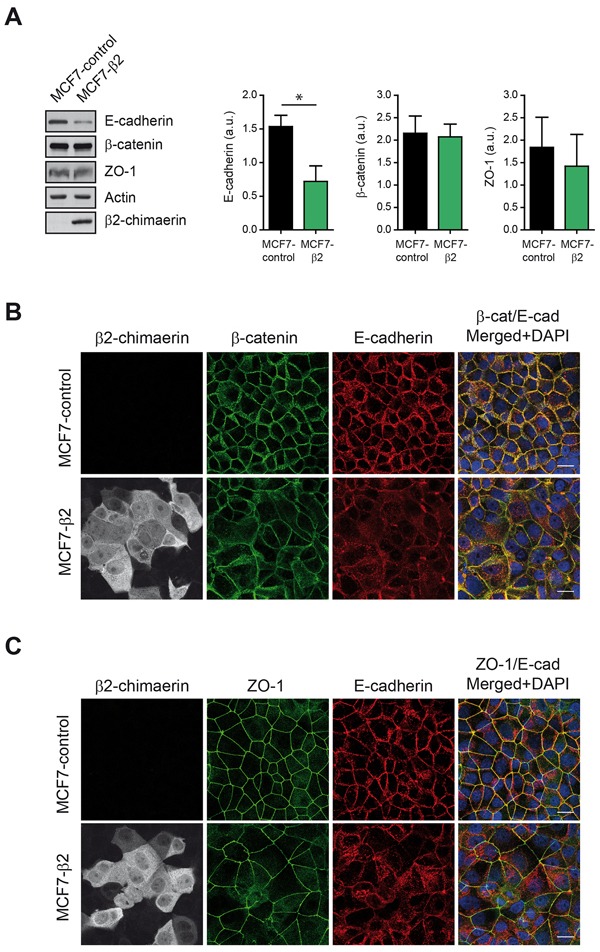
β2-chimaerin regulates E-cadherin levels but has no effect on β-catenin and ZO-1 **A.** Western blot analysis of E-cadherin, β-catenin, and ZO-1 expression in MCF7-control and MCF7-β2 cells. Densitometric analysis normalized to the actin expression is shown in the histograms. Results are presented as means ± s.e.m. (n = 3) (*P < 0.05, unpaired two-tailed Student's t-test). **B-C.** Immunofluorescence images of β-catenin and E-cadherin (B), or ZO-1 and E-cadherin (C), in MCF7-control and MCF7-β2 cells. Panels show representative confocal immunofluorescence images from one of three independent experiments. Scale bars: 20 μm.

We next evaluated the expression of E-cadherin by Western blot analysis (Figure 2A). We found that cells expressing β2-chimaerin had a statistically significant reduction in the levels of E-cadherin compared to control cells. However, the expression of the AJ-associated β-catenin and the tight junction (TJ) protein ZO-1 remained unchanged (Figure 2A). Similarly, immunofluorescence staining revealed that recruitment of β-catenin and ZO-1 to cell junctions was not severely compromised in MCF7-β2 cells (Fig 2B and 2C). These results suggest that β2-chimaerin predominantly alters E-cadherin expression but has no major effect on other AJ or TJ components.

### β2-chimaerin regulates cell-cell adhesion and differentially affects migration and invasion depending on the cell context

**Figure 3:**
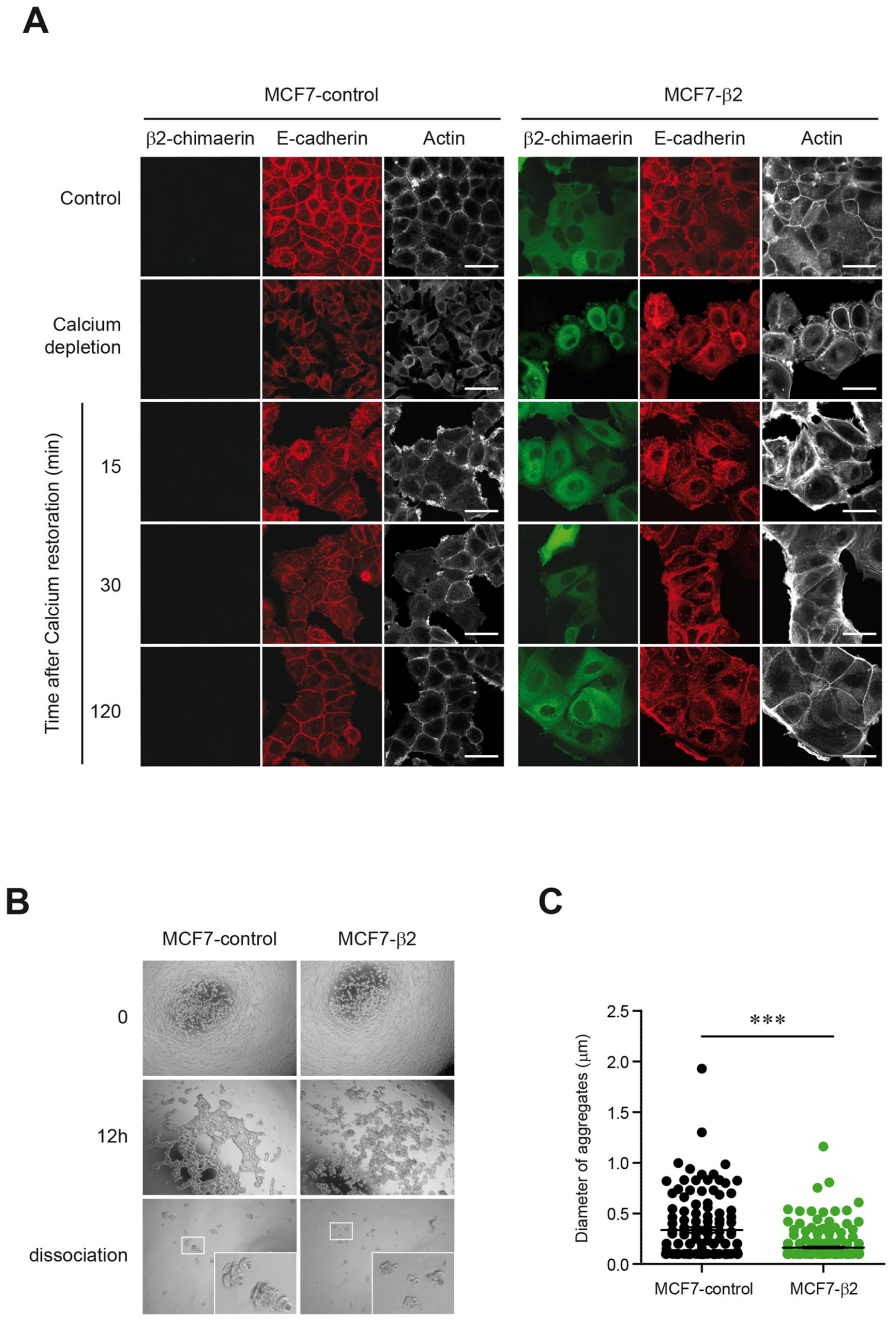
Expression of β2-chimaerin regulates cell-cell adhesion in MCF7 cells **A.** Confocal immunofluorescence images of E-cadherin and actin staining of MCF7-control and MCF7-β2 cells subjected to a calcium switch assay. Panels show representative images from one of three independent experiments. Staining of cell grown in control media is also shown (upper panels). Scale bars: 40 μm. **B-C.** Hanging drop aggregation assay in MCF7-control and MCF7-β2 cells (B) Representative images (4×) from each experimental condition (a higher magnification of cell aggregates after dissociation is shown in the inset) (C) Scatter plot of the size of aggregates from two independent experiments (***P < 0.001, unpaired two-tailed Student's t-test).

E-cadherin is an important regulator of epithelial cell-cell adhesion by contributing to the formation and maintenance of adherens junctions (AJs) [29]. Because expression of β2-chimaerin reduced the levels of E-cadherin in MCF7 cells we first investigated whether β2-chimaerin influences AJs assembly. To this end, we conducted a calcium switch assay, which involves the disruption of E-cadherin-based cell-cell adhesion by removal of extracellular calcium, followed by the reassembly of cell-cell contacts after restoration of normal calcium levels [30]. We observed that, as expected, MCF7-β2 cells showed reduced levels of E-cadherin at AJs throughout junction formation. However, these cells formed apparently normal AJs after 2h of calcium repletion (Figure 3A). Furthermore, the remodeling of peripheral actin 2h after calcium restoration was not compromised and resulted in a similar extent of accumulation at cell-cell boundaries as in control cells.

We next performed a hanging drop assay that measures formation of junctions and their resistance to shearing force [31]. After 12 h of incubation of individual cells in hanging drops, both MCF7-control and MCF7-β2 cells formed large interconnected clusters with a web-like organization, but the aggregates of MCF7-β2 cells appeared much more loosely arranged and less compacted (Figure 3B). Upon trituration, aggregates dispersed into significantly smaller clusters in the β2-chimaerin expressing cells (Figure 3B, 3C). Together, these results indicate that β2-chimaerin does not impede the initial formation and the dynamics of AJ assembly but reduces the strength of cell-cell adhesion.

**Figure 4:**
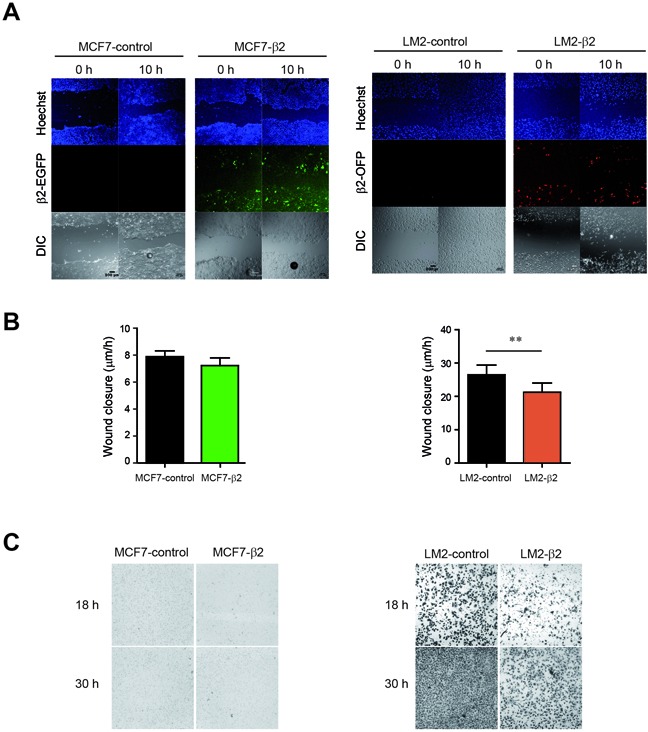
β2-chimaerin differentially affects cell migration and invasion in epithelial or mesenchymal-like cells **A-B.** MCF7-control, MCF7-β2, LM2-control and LM2-β2 cell lines were subjected to a wound healing assay. **A.** Representative images showing the wound closure at two different time points. Nuclei were stained with Hoechst to corroborate that equal number of cells were used in each case. **B.** Quantification of the relative migration of control versus β2-chimaerin expressing cells. Results are presented as means ± s.e.m (n=7) (**P < 0.01, unpaired two-tailed Student's t-test). **C.** Invasion of MCF7-control, MCF7-β2, and LM2-control and LM2-β2 cells was assessed using the Boyden chamber assay in the presence of Matrigel-coated filters. Images show crystal violet-stained migrated cells present at the lower face of the transwell membranes and are representative of one out of three different experiments.

Given that a decrease of E-cadherin is associated with increased metastatic potential of tumor cells [32], our result showing that β2-chimaerin reduces E-cadherin is paradoxical, since it had been reported the inhibition of migration and invasion of breast cancer cells by β2-chimaerin [22]. This discrepancy could be partially attributed to the use of non-epithelial breast cancer cells in the previous study. In order to clarify this issue, we compared the effect of β2-chimaerin in the migration and invasion of MCF7 and LM2 cells, a mesenchymal-like breast cancer cell line that lacks E-cadherin expression. To this end, we generated LM2 cells stably expressing β2-chimaerin fused to orange fluorescent protein (β2-chimaerin-OFP). Similar to MCF7-β2 cells, the expression of β2-chimaerin in LM2 cells was ~10-fold increase over the endogenous β2-chimaerin in normal human breast tissue (Supplementary Figure S1). By performing wound healing assays in these cell lines, we found that β2-chimaerin significantly inhibited cell migration in LM2 cells, while had no effect on MCF7 cells (Figure 4A, 4B). Additionally, β2-chimaerin clearly reduced invasion of LM2 cells in transwell assays, whereas no invasion was observed in either MCF7-control, or MCF7-β2 cells (Figure 4C).

Collectively, these results indicate that β2-chimaerin regulates at least two different cytoskeleton-dependent processes in breast cancer cells, migration and adhesion, but the final output of this regulation is highly dependent on the cell type. This raises an important question as for the exact role of β2-chimaerin in breast cancer, a pathology that arises from epithelial cells that switch to mesenchymal cells during cancer progression. To address this question we decided to study the role of β2-chimaerin in breast cancer development and progression *in vivo*.

### Downregulation of β2-chimaerin *in vivo* accelerates tumor onset but delays tumor progression

In light of data reporting the downregulation of β2-chimaerin in breast cancer [20], and based on mechanistic insights showing that β2-chimaerin and Rac1 regulate cell proliferation, migration and invasion downstream of ErbB receptors [24, 33, 34], we selected the MMTV-Neu mouse model, which develops breast cancer due to overexpression of the Neu (ErbB2/Her2) receptor, to study the effects of β2-chimaerin deficiency *in vivo*. To corroborate that our studies in MCF7 cells showing that β2-chimaerin decreases E-cadherin can be extrapolated to this model, we first confirmed that β2-chimaerin also downregulates E-cadherin protein levels in BT474 cells, a breast cancer epithelial cell line that overexpresses the ErbB2 receptor (Supplementary Figure S1 and S2).

**Figure 5:**
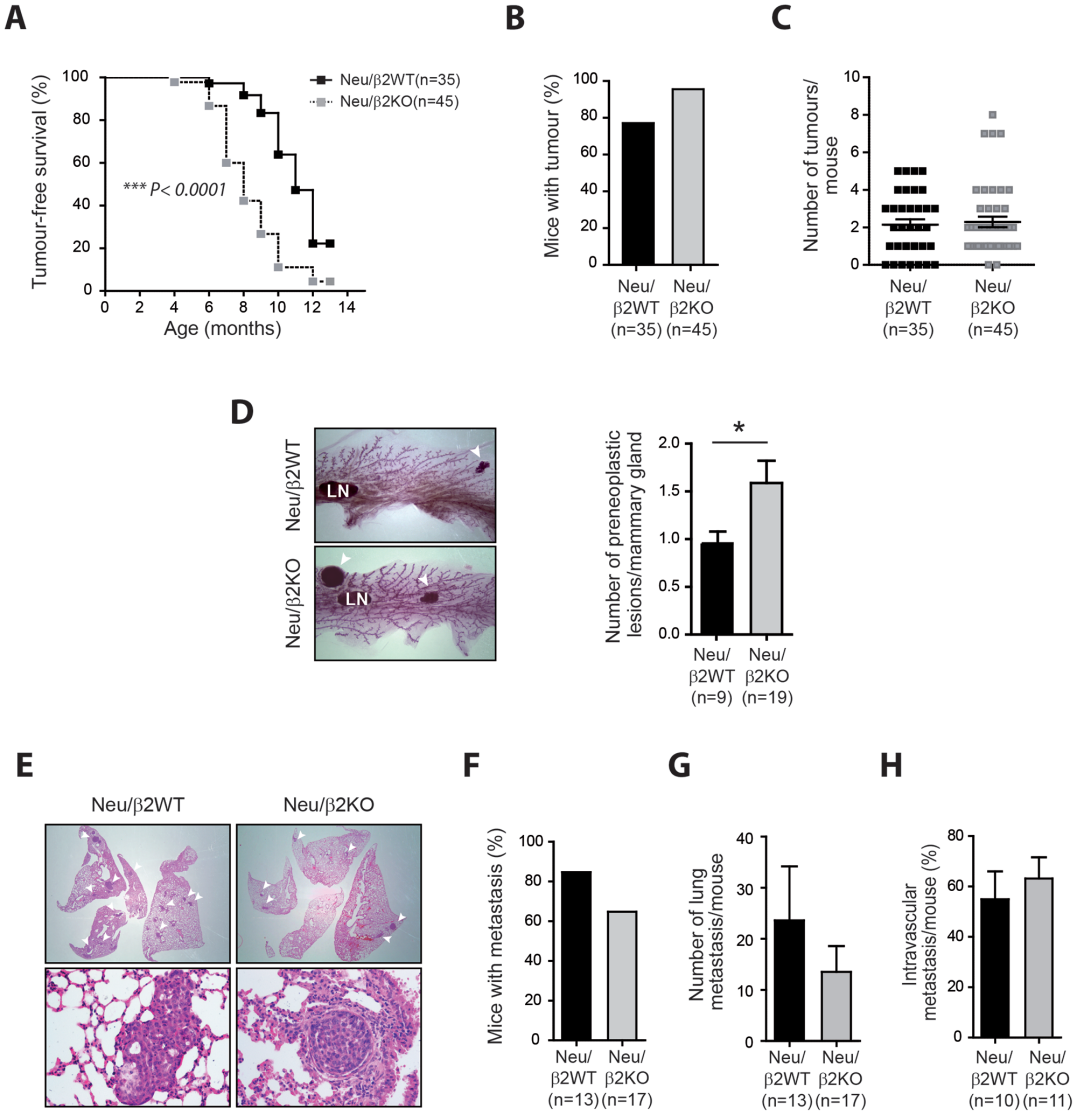
β2-chimaerin deficiency accelerates mammary tumor onset but delays tumor progression *in vivo* **A.** Kaplan-Meier plots of tumor-free survival. Tumor latency was significantly reduced in Neu/β2KO mice (***P < 0.0001, log rank test). **B.** Mammary tumor incidence (*P* = 0.16). **C.** Scatter plot showing the number of mammary tumors per mouse (*P* = 0.95). **D.** Representative whole mounts of mammary glands showing preneoplastic lesions. Arrowheads: preneoplastic lesions. LN: lymphatic node. The number of preneoplasias per tumor-free mammary gland is shown in the histograms (*P < 0.05). **E-H.** Analysis of lung metastasis in Neu/β2WT and Neu/β2KO mice. (E) Representative H&E staining of lung sections showing metastasis. Top: arrowheads indicate metastatic foci in the lung (4×). Bottom: image showing metastasis invading the lung parenchyma (left) or inside a blood vessels (right) (20×). **F.** Incidence of lung metastasis (*P* = 0.24). **G.** Number of metastatic foci per mice (*P* = 0.397). **H.** Percentage of intravascular metastasis (*P* = 0.479). Statistical significance in B-D and F-H was assessed by Mann Whitney *U*-test. Results are presented as means ± s.e.m

To generate β2-chimaerin-deficient MMTV-Neu mice, we obtained mice with a gene-trap insertion in the *Chn2* gene that eliminates β2-chimaerin expression (Supplementary Figure S3) and crossed them with MMTV-Neu mice to generate two cohorts of females; MMTV-Neu^+/+^/*Chn2*^+/+^ (thereafter named Neu/β2WT) and MMTV-Neu^+/+^/*Chn2*^−/−^ (thereafter named Neu/β2KO). Neu/β2KO females nursed their offsprings to maturity and showed no gross defects on the mammary gland architecture, suggesting that loss of β2-chimaerin does not impair mammary gland function (Supplementary Figures S3D and S4). Tumorigenesis studies revealed that mammary tumor development was significantly accelerated in Neu/β2KO mice as determined by Kaplan-Meier analysis (median time for tumor onset 8 months for Neu/β2KO mice versus 11 months for Neu/β2WT mice, *P* < 0.0001) (Figure 5A). Tumor incidence and tumor multiplicity did not show significant differences between both genotypes (Figure 5B and 5C), although tumor incidence was slightly higher in Neu/β2KO females (96.6% versus 77.1%) (Figure 5B). Additionally, the mammary glands of Neu/β2KO females exhibited a significant increase in the number of preneoplastic lesions when compared with Neu/β2WT females (*P* < 0.05) (Figure 5D). These results indicate that more tumors are initiated in β2-chimaerin-deficient mice during the time of primary tumor growth. Together, these data represent the first animal model that reveals a tumor suppressor role for β2-chimaerin in Her2/Neu induced primary mammary tumor formation, thus undoubtedly demonstrating a function for this protein in breast carcinogenesis.

We next evaluated whether there were differences in the metastatic potential between tumors that developed in Neu/β2WT and Neu/β2KO mice (Figure 5E–5H). We found no statistically significant difference in the incidence of lung metastasis or number of metastatic foci per mice between Neu/β2WT and Neu/β2KO mice (84.6% versus 64.7%, *P* = 0.24 and 23.6 ± 10.6 versus 13.5 ± 5.0, *P* = 0.4 respectively) (Figure 5F and 5G). Additionally, similar percentages of pulmonary metastasis were confined to the lung vasculature in mice of both genotypes (Figure 5H). Thus, despite previous studies in cell lines showing that β2-chimaerin inhibits processes involved in the metastatic cascade such as migration and invasion [22–24], our data demonstrate *in vivo* that downregulation of this protein does not result in increased metastasis.

### Deletion of β2-chimaerin in MMTV-Neu mice correlates with lower mammary tumor grade

**Figure 6:**
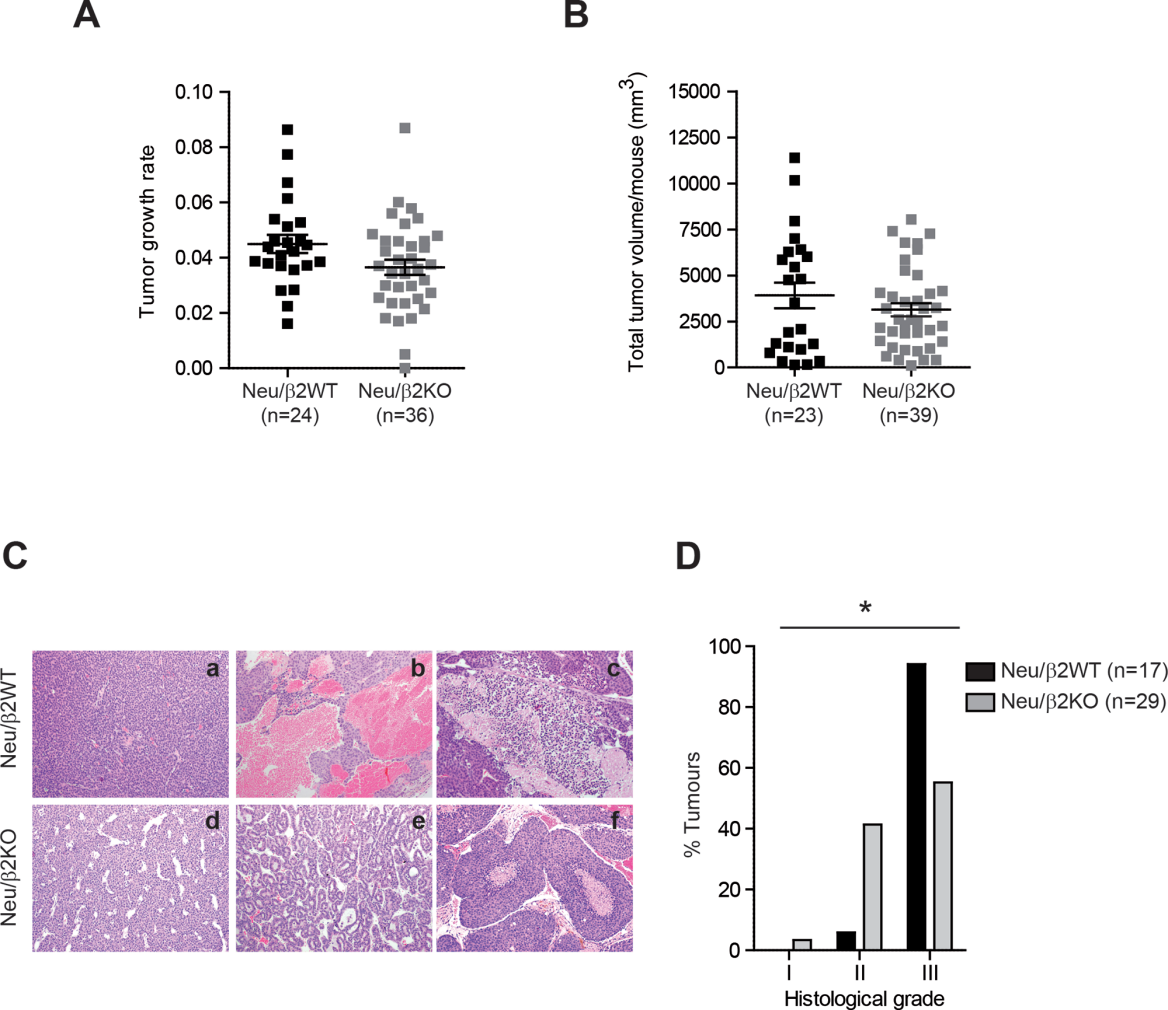
β2-chimaerin deficiency correlates with lower mammary tumor grade **A.** Tumor growth rate in Neu/β2WT and Neu/β2KO mice. Tumor growth was analyzed for 6 weeks after tumor onset by weekly measurement of tumor volume. Data were represented on a semilogaritmic scale and fitted by linear regression. Slopes for each growth curve are represented (*P* = 0.089, Mann Whitney *U*-test). **B.** Tumor burden in Neu/β2WT and Neu/β2KO mice (*P* = 0.74, Mann Whitney *U*-test). **C.** Representative images of H&E staining of Neu/β2WT and Neu/β2KO tumors (10×) showing poorly differentiated carcinomas (a, d), a moderately differentiated carcinoma (e), a tumor with hemorrhagic areas (b), and tumors with necrotic areas (c, f). **D.** Grade distribution of tumors according to the modified Elston and Ellis grading system (*P < 0.05, two tailed Fisher's exact test).

To further evaluate the role of β2-chimaerin in tumor progression we compared the growth rates and tumor burden of Neu/β2WT and Neu/β2KO mammary tumors (Figure 6A and 6B). We found no statistically significant differences in these tumor features, although Neu/β2KO tumors showed a trend toward slower growth (*P* = 0.089) (Figure 6A). We next compared the histopathologic features of mammary tumors from both cohorts (Fig 6C and 6D). We found a significant difference in tumor grade distribution among tumors from both genotypes (*P* = 0.015). 94.1% of the tumors from Neu/β2WT mice were poorly differentiated carcinomas with high histologic grade. However, in Neu/β2KO mice only 55.2% of the tumors were of high grade (Figure 6D). Additionally, large areas of necrosis and hemorrhage, which associates with tumor aggressiveness, were more frequently observed in tumors from control mice (88.2% in Neu/β2WT versus 43.5% in Neu/β2KO mice). Collectively, these data indicate that, despite the reduction in latency, tumors from Neu/β2KO mice exhibit a less aggressive behavior.

### Deletion of β2-chimaerin in MMTV-Neu mice results in increased Rac activity without affecting proliferation, cell death or angiogenesis

**Figure 7:**
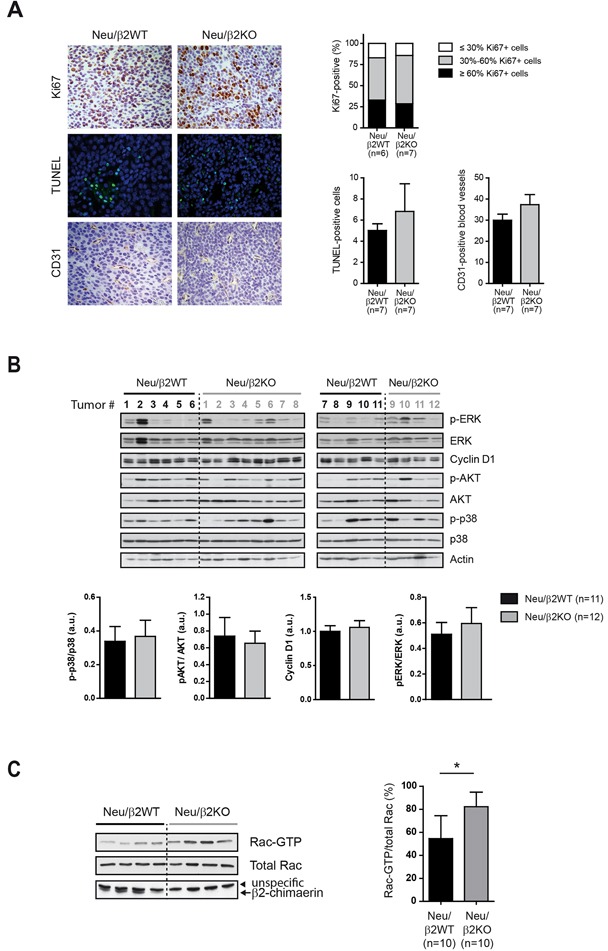
Deletion of β2-chimaerin in MMTV-Neu mice leads to Rac1 activation in tumors but has no effect in proliferation **A.** Proliferation, apoptosis and angiogenesis in the tumors of the indicated genotypes. Representative images of Ki67, TUNEL and CD31 staining are shown. Histograms show the percentage of Ki67-positive cells (*P* = 0.99, two tailed Fisher's exact test), TUNEL-positive cells (*P* = 0.90, Mann Whitney *U-*test), and CD31-positive blood vessels (*P* = 0.469, Mann Whitney *U-*test). **B.** Western blot analysis of the expression and phosphorylation status of the indicated proteins in tumor lysates from mice of the indicated genotypes. Densitometric analyses are shown in the histograms. P-p38, pAKT and pERK levels were normalized to the corresponding total protein (*P* = 0.93, *P* = 0.92, and *P* = 0.83 respectively, Mann Whitney *U-*test). Cyclin D1 protein level was normalized to actin (*P* = 0.78, Mann Whitney *U-*test). **C.** Rac1-GTP levels in mouse tumors from the indicated genotypes. A representative pull-down assay is shown. Histograms show the quantification of Rac1-GTP levels normalized to the total Rac1 (*P <0.05, Mann Whitney *U-*test). Results are presented as means ± s.e.m

To investigate the possible mechanisms by which β2-chimaerin ablation in MMTV-Neu mice influences breast tumor development and progression, we examined proliferation, cell death and angiogenesis by Ki-67, TUNEL and CD31 staining respectively. We found no statistically significant differences in these markers between tumors from Neu/β2WT and Neu/β2KO mice (Figure 7A). Additionally, Western blot analysis revealed no consistent differences in the activation or expression of ERK, cyclin D1 or AKT, main signaling effectors activated by overexpression of Neu that drive cell proliferation and resistance to apoptosis [35–37] (Figure 7B). The phosphorylation status of the p38 MAP kinase, a regulator of cell death involved in mammary tumorigenesis [38] was also unaffected by β2-chimaerin ablation (Figure 7B).

β2-chimaerin functions are mediated through Rac inactivation [14, 20]. To corroborate that Rac activity was increased due to β2-chimaerin deletion and rule out compensatory effects by other GAP proteins, we performed Rac-GTP pull-down assays on tumor lysates from Neu/β2WT and Neu/β2KO mice. This analysis revealed a statistically significant increase in the levels of active Rac1 in Neu/β2KO tumors (*P* < 0.05) (Figure 7C). Together, these results suggest that loss of β2-chimaerin does not affect proliferative, apoptotic and angiogenic pathways in mammary tumors despite increasing Rac activity.

### Loss of β2-chimaerin favors the maintenance of the epithelial state of breast tumors

**Figure 8:**
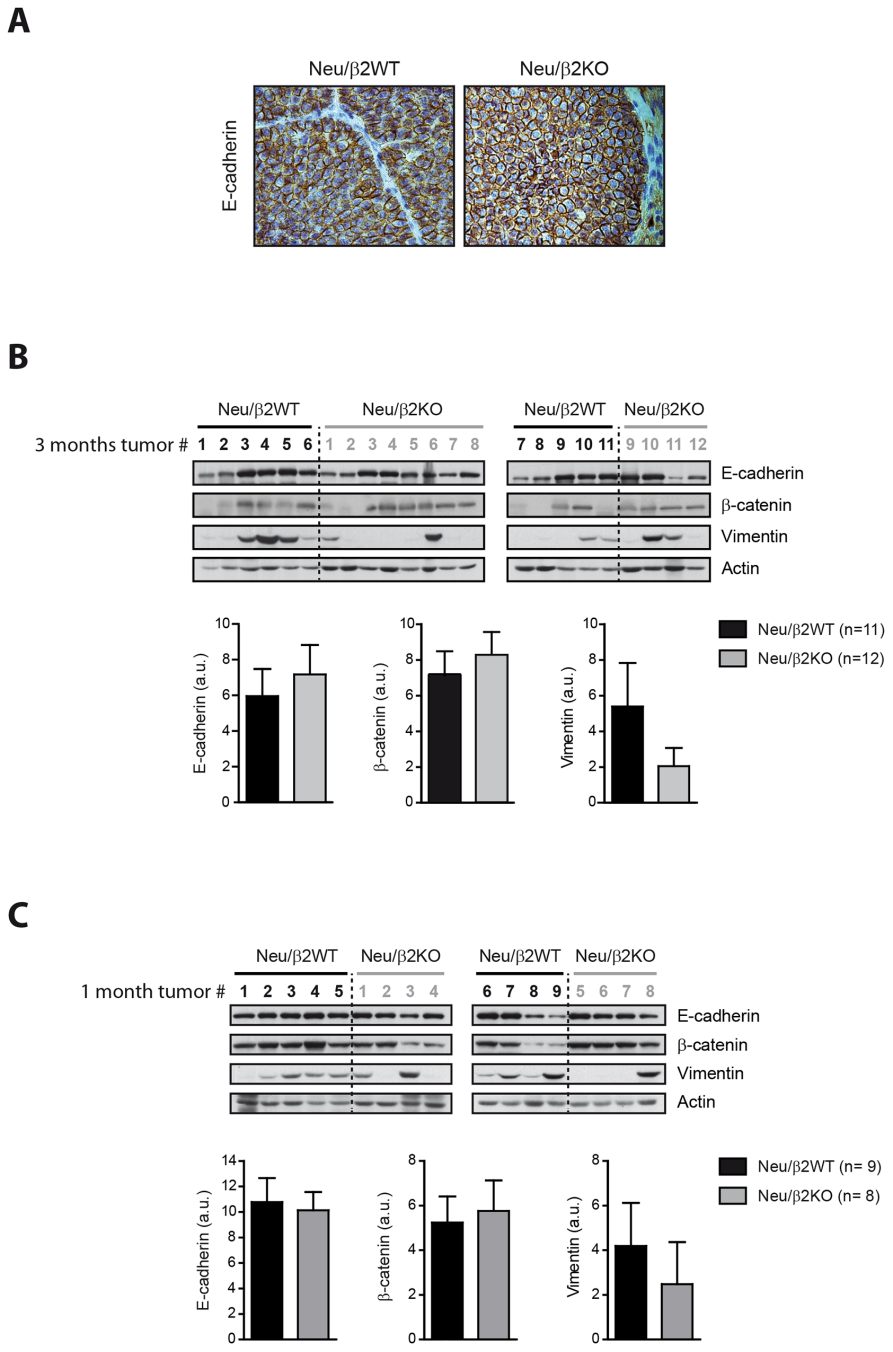
Deletion of β2-chimaerin in MMTV-Neu mice favors the maintenance of the epithelial state of breast tumors **A.** Representative images of E-cadherin staining in tumors of the indicated genotypes collected after three months from tumor onset. **B-C.** Western blot analysis of the expression of E-cadherin, β-catenin and vimentin in tumor lysates from Neu/β2WT and Neu/β2KO mice collected after 3 months from tumor onset (B) (same tumors as in Figure 6B), or after 1 month from tumor onset (C). Densitometric analysis of the expression of each protein normalized to actin expression is shown in the histograms. In B: *P* = 0.69 for E-cadherin, *P* = 0.87 for β-catenin and *P* = 0.17 for vimentin expression; in C: *P* = 0.81 for E-cadherin, *P* = 1.00 for β-catenin and *P* = 0.07 for vimentin (Mann Whitney *U*-test). Results are presented as means ± s.e.m.

Given that we found that expression of β2-chimaerin in breast cancer epithelial cells reduces E-cadherin, we asked whether ablation of β2-chimaerin in MMTV-Neu mice could delay cancer progression by increasing E-cadherin levels. IHC analysis showed strong E-cadherin staining in tumors from both, Neu/β2WT and Neu/β2KO mice, which mainly localized to the cell-cell junctions (Figure 8A). Additionally, Western blot analysis showed similar E-cadherin content in tumors from both genotypes (Figure 8B). Similarly, the levels of the epithelial marker β-catenin were approximately equal in tumors from both cohorts (Figure 8B). We subsequently analyzed the expression of vimentin, a marker of epithelial-to-mesenchymal transition (EMT), and found that more tumors from Neu/β2WT mice expressed this protein (8/11 versus 4/12 in Neu/β2KO Figure 8B). Similar results were observed at an earlier stage of breast tumor development (Figure 8C). Therefore, although the overall E-cadherin expression in MMTV-Neu mice tumors was not greatly affected by loss of β2-chimaerin, our data suggests that β2-chimaerin ablation favors the maintenance of the epithelial state of breast cancer cells, thus delaying EMT in MMTV-Neu tumors.

### β2-chimaerin expression predicts relapse-free survival in human breast cancer and inversely correlates with E-cadherin expression in the Her2+ subtype

**Figure 9:**
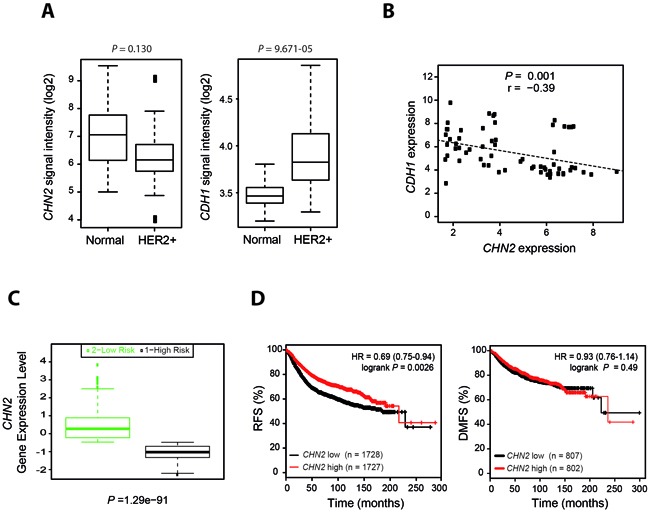
Reduced β2-chimaerin expression in human breast cancer inversely correlated with E-cadherin expression and is associated with reduced relapse-free survival **A.** *CHN2* and *CDH1* expression values in normal breast tissue and untreated Her2+ breast tumors (data from the GSE29431 dataset). The *P* values shown were calculated by the Student's t test followed by the Benjamini-Hochberg correction **B.** Inverse correlation between *CHN2* and *CDH1* expression in untreated Her2+ breast tumors. Data were retrieved from the GSE29431 and GSE65216 datasets and were plotted by expression value. Data analysis was performed using the Pearson's correlation test. **C.** Association of β2-chimaerin expression with survival. Box plots were generated by SurvExpress and show the relative *CHN2* mRNA expression levels of breast carcinoma samples from TCGA. Low- and high-risks groups are shown in green and black, respectively. The *P* value results from a Student's t test analysis. **D.** Kaplan-Meier plots of relapse-free survival (RFS) and distant metastasis-free survival (DMFS) of breast cancer patients stratified by expression of the *CHN2* gene; high (red) or low (black). Statistical significance was assessed by the log-rank test. **P < 0.01 for RFS analysis. In all studies the probe set for *CHN2* was 213385_at and for *CDH1* was 201130_s_at.

To evaluate the relevance of our experimental findings in the pathogenesis of human breast cancer, we first analyzed the expression levels of the β2-chimaerin and E-cadherin genes (*CHN2* and *CDH1* respectively) in Her2+ tumors and in normal breast tissue using microarray datasets that are publicly available. Given that recent studies suggests that Rac1 is involved in drug resistance in Her2+ patients [39, 40], only untreated patients were included in the study. We observed that *CHN2* expression was reduced in Her2+ samples when compared with normal breast tissue, whereas the expression of *CDH1* was increased (Figure 9A). In a further analysis, we detected an inverse correlation between the expressions of both genes in Her2+ patients (Figure 9B). Next, we investigated whether β2-chimaerin expression correlates with survival. Using the SurvExpress tool to interrogate the TCGA (The Cancer Genome Atlas) database we found that *CHN2* expression was significantly reduced in the high-risk population (Figure 9C). Additionally, we examined the prognostic value of *CHN2* expression in a large clinical microarray database of human breast tumors [41]. We found that low *CHN2* expression levels in unstratified breast cancer patients significantly correlated with reduced relapse-free survival (*P* < 0.001). However, distant-metastasis free survival was similar regardless of the levels of *CHN2* expression (Figure 9D). Overall, these clinical data are in agreement with our results in Neu/β2KO mice and support a tumor suppressor function of β2-chimaerin in tumor initiation.

## DISCUSSION

Our study here unequivocally establishes for the first time the *in vivo* relevance of the Rac-GAP β2-chimaerin in breast carcinogenesis. We found that, in agreement with its proposed role as tumor suppressor [20], deletion of β2-chimaerin in MMTV-Neu mice increases their susceptibility to develop breast tumors, as tumor latency was significantly reduced and mice had a higher incidence of preneoplasic lesions in the mammary glands. Unexpectedly, however, β2-chimaerin ablation delayed cancer progression since most tumors from β2-chimaerin KO mice were of lower grade.

Downregulation of β2-chimaerin has been shown to promote the proliferation in different epithelial cells [26, 42]. Additionally, studies in breast cancer cells demonstrated that β2-chimaerin inhibits proliferation downstream of the ErbB2 receptors, an effect entirely dependent on its Rac-GAP activity [20, 24, 25]. Therefore, we had hypothesized that deletion of β2-chimaerin would increase proliferation in Neu-induced tumors. However, our data revealed that tumors from β2-chimaerin KO mice had proliferation rates similar to those from control mice, despite having higher Rac1 activity. One possible explanation for this discrepancy lies in the different levels of expression of the upstream receptor in these experimental models. While the cell-based studies were largely performed under conditions of normal growth factor signaling, the high overexpression of the ErbB2 receptor in tumors on the MMTV-Neu model is sufficient to strongly activate mitogenic signaling, and thus, may override β2-chimaerin deficiency (41).

We have uncovered a new function of β2-chimaerin in the maintenance of cell junctions that may explain the delay in cancer progression in β2-chimaerin-deficient MMTV-Neu mice by the impediment of the disassembly of cancer cells. This finding is supported by our results showing that expression of β2-chimaerin in MCF7 and BT474 cells reduces E-cadherin levels, resulting in the weakening of intercellular adhesive strength. Of note, our results parallel those found when Rac1 is inhibited in epithelial cells, which also induces the loss of E-cadherin at cell-cell contacts and diminishes contact strength [26, 30, 31, 43]. Besides, Rac1 inhibition results in larger cell diameters [43], an effect that we also observed in MCF7 cells expressing β2-chimaerin and can be explained by a reduced tension due to the loosening of intercellular contacts [44]. Thus, our results are consistent with impairment on cell-cell adhesion by β2-chimaerin expression due to inhibition of Rac1. In support of this hypothesis, a previous study described that downregulation of the *Drosophila* chimaerin orthologue affects the stability of adherens junctions, an effect also attributed to increased activation of Rac in absence of chimaerin [26].

Although reduction of E-cadherin leads to the acquisition of migratory and invasive properties of cancer cells, we have not observed any increase in the migration and the invasion of MCF7 cells expressing β2-chimaerin. This can be explained by the fact that cells still maintain a significant amount of E-cadherin and other components of AJs as well as TJs are unaffected. These results are in apparent contradiction with previous studies reporting the inhibition of migration and invasion of breast cancer cells by β2-chimaerin [22, 24]. These studies, however, were performed in non epithelial cells [22], or in MCF7 cells subjected to chemotactic stimulus and experimental conditions that involve the disruption of cell–cell adhesion [24]. Furthermore, our results in mesenchymal breast cancer cells further corroborate that β2-chimaerin can inhibit migration and invasion in a non-epithelial context. Hence, our data, together with those of previous studies, suggest a differential role for β2-chimaerin in cell migration and invasion depending on the cell type and the cellular context.

Breast cancer is a pathology that originates from epithelial cells which acquire a mesenchymal phenotype during cancer progression. Considering the above results, loss of β2-chimaerin could either favor or delay the metastatic potential of breast cancer cells depending on their phenotype. Our *in vivo* analysis revealed that, indeed, ablation of β2-chimaerin hinders tumor progression. We observed a clear delay in the EMT in absence of β2-chimaerin, since significantly less Neu-induced tumors from β2-chimaerin KO mice showed expression of the mesenchymal marker vimentin, while most of the control mice expressed this marker. We could not establish a clear correlation between β2-chimaerin and E-cadherin expression in our *in vivo* model, as we observed in breast cancer cell lines, since tumors from both mice cohorts showed high levels of E-cadherin. Nevertheless, since changes in E-cadherin expression during metastatic progression are transient and confined to the invasive tumor front [45], we cannot rule out differences in this protein that we have not detected in our analysis. Importantly, the bioinformatics analyses of human breast cancer databases show an inverse correlation between E-cadherin and β2-chimaerin expression, which supports a role of β2-chimaerin in the regulation of E-cadherin *in vivo*. Interestingly, times to relapse are shorter in breast cancer patients with low β2-chimaerin expression than in patients with high expression but both groups of patients develop metastasis at similar times, suggesting that loss of β2-chimaerin may also delay metastasis in human breast cancer.

In summary, this study delineates for the first time the *in vivo* function of β2-chimaerin in breast cancer and provides further insight into the mechanisms by which this protein influences tumor progression.

## MATERIALS AND METHODS

### Ethics statement

Investigation has been conducted in accordance with the ethical standards, according to the Declaration of Helsinki and according to national and international guidelines. Animal care and work protocols were approved by the Bioethics Committees of the University of Valladolid and CSIC.

### Cell lines

MCF7 and BT474 cells were provided by Dr. Pandiella (CIC, Salamanca, Spain) who authenticated the cells. The MDA-LM2-4175 (LM2) cell line was donated by Dr. Massagué (Memorial Sloan-Kettering Cancer Center, NY, USA). Cells were grown at 37°C and 5% CO_2_ in DMEM supplemented with 10% fetal bovine serum (FBS), 1% L-glutamine and 100 units/ml penicillin and streptomycin (Gibco). MCF7 and BT474 cells stably expressing β2-chimaerin were generated by transfection of the pEGFP-N1-CHN2 vector encoding EGFP-tagged β2-chimaerin. The LM2 cell line contains a GFP reporter, and thus, to generate LM2 cells stably expressing β2-chimaerin cells were transfected with a vector encoding Orange-fluorescent-protein-tagged β2-chimaerin (pOFP-N1-CHN2). 48 h post-transfection, cells were selected with 500 μg/ml geneticin (Gibco), and then sorted by FACS to collect fluorescent cells expressing β2-chimaerin (MCF7-β2, BT474-β2 and LM2-β2). Non-fluorescent cells were also collected and used as control (MCF7-control, BT474-control and LM2-control).

To knock-down β2-chimaerin expression, MCF7-β2 cells were infected with lentiviral shRNA targeting the β2-chimaerin sequence (CGTACACAAACAGTGTTCCAA) or scrambled shRNA as control. After selection in 2 μg/ml puromycin, EGFP-β2-chimaerin knock-down was confirmed by loss of GFP fluorescence in resistant cells.

### Quantitative RT-PCR

Quantitative RT-PCR analysis was performed with the QuantiTect One step SYBR Green PCR Kit (Qiagen), using 150 ng of total RNA from the cell lines (isolated with TRIzol, Invitrogen) and from human breast tissue (Agilent). Primers for β2-chimaerin were: 5′-GCAGGCGGATGAGCTTCTT-3′ (forward) and 5′-CTCACCCACAAAGTGTTTCCC-3′ (reverse). Primers for GAPDH were: 5′-TGCACCACCAACTGCTTAG-3′ (forward) and 5′-GGATGCAGGGATGATGTTC-3′ (reverse). Quantitative RT-PCR was performed on a Light Cycler® 480 Real-Time PCR System (Roche Diagnostics). Relative β2-chimaerin expression was calculated using a standard 2^−ΔΔCT^method.

### Immunofluorescence

For E-cadherin, β-catenin, ZO-1 and actin staining, equal number of MCF7-β2 and MCF-7 cells were seeded on coverslips and grown to confluence. Cells were fixed with 3.7% formaldehyde, permeabilized in 0.2% Triton X-100 for 10 min and incubated in blocking solution containing 1% BSA and 100 mM glycine in phosphate-buffered saline (PBS). Cells were then labelled with the following primary antibodies: anti-E-cadherin (610181, BD Transduction Laboratories), anti β-catenin (D10A8, Cell Signaling) or anti ZO-1 (D7D12, Cell Signaling). Actin was labelled with Alexa-647 phalloidin (Cell Signaling) or rhodamine-phalloidin (Invitrogen). For vinculin staining cells were seeded onto coverslips coated with collagen IV (30μg/ml), processed as indicated above and stained with anti-vinculin (V9264, Sigma). Nuclei were stained with DAPI (1μg/ml). Cells were imaged using a laser scanning confocal microscope (Leica TCS SP5 or Olympus Fluoview 1000) and confocal micrographs were processed with ImageJ (NIH).

### Cell-cell adhesion assays

For calcium switch experiments, cells were incubated overnight in calcium-free DMEM (PAN Biotech) and then switched back to standard DMEM growth media. Cells were fixed at the indicated times, processed for E-cadherin and actin staining as indicated above and analyzed by confocal microscopy. Hanging drop aggregation assays were performed as described previously [31]. In brief, MCF7-β2 and MCF7-control cells were trypsinized, centrifuged, and resuspended as single-cell suspensions at 2 × 10^5^ cells/ml. 30 μl drops of cell suspension were pipetted into the inside surfaces of 10 cm culture dish lids. After overnight incubation, cells were subjected to shear force by passing them 10 times through a standard pipet tip. Cells were photographed using a microscope (Nikon Eclipse TS100) equipped with a digital camera (Nikon DS-5M). Cell cluster sizes were analyzed using ImageJ.

### Migration and invasion assays

Cell migration was analyzed in wound healing assay. To this end, 2 × 10^5^ control and β2-chimaerin expressing MCF7 or LM2 cells were seeded in 24-well plates. When cultures reached confluency, wounds were created by scraping with 200-μl plastic tips across the cell monolayer. The cell culture medium was then removed, wells were washed with PBS to remove detached cells and then 1 ml of DMEM media without serum was added to each well. Wound closure was monitored with a confocal microscope (Olympus Fluoview 1000) and images were captured every 5 min with a 10x objective. The wound healing rate was calculated using ImageJ.

Invasion assays were performed using 8-μm pore invasion chambers (Costar) that were coated with 100 μl Matrigel (BD Biosciences) in DMEM (1:20). Control and β2-chimaerin expressing MCF7 and LM2 cells were trypsinized, suspended in DMEM and plated in the upper chamber (2.5 × 10^5^ cells/well). The lower chamber was filled with DMEM containing 10% FBS as chemoattractant. After 18-30 h incubation, the wells were washed with PBS and invasive cells located on the lower side of the chamber were stained with crystal violet, air-dried and photographed.

### Mice strains

All mice were housed at the Animal Research Facility of the University of Salamanca and the University of Valladolid. Mice containing a gene-trap insertion in the β2-chimaerin gene (*Chn2*) were obtained from Lexicon Genetics (Woodland, TX, USA) and were on a mixed C57Bl/6/129/SvEvBrd background. The gene trap was inserted in the intron 1 of the *Chn2* gene that truncates the β2-chimaerin transcript immediately after the first coding exon. The gene trap does not affect the β1-chimaerin transcript which initiates from a promoter located upstream of exon 7. Genotyping was performed by PCR of mouse tail DNA according to Lexicon's recommendations. Expression of β2- and β1-chimaerin transcripts were analyzed by RT-PCR using the following primers: 5′-CGTGGAAGGTGCCTACATCC-3′ (forward) and 5′-TTCACCATCTCTGTCAAACGCC-3′ (reverse) for amplification of β2-chimaerin, and 5′-GCTATGGTT CGGAAGTCCAAGC-3′ (forward) and 5′-TTCACCATCT CTGTCAAACGCC-3′ (reverse) for amplification of β1-chimaerin. The p36b4 with gene was used as control and was amplified with the following oligos: 5′-GTGTTTGACAACGGCAGCATT-3′ (forward) and 5′-TTGATGATGGAGTGT GGCACC-3′ reverse).

FVB/N-Tg (MMTV-Neu) 202Mul/J mice, which are FVB mice carrying the ErbB2 protooncogene under the control of the MMTV 3′promoter [46], were originally from the Jackson Laboratory (Bar Harbor, ME, USA). To generate β2-chimaerin-defficient MMTV-Neu mice, β2-chimaerin KO mice (*Chn2*^−/−^) were first backcrossed to the FVB background for 4 generations. Homozygous *Chn2*^−/−^ mice were crossed with MMTV-Neu mice to generate double heterozygous mice that were then intercrossed to generate two cohorts of females; MMTV-Neu^+/+^/*Chn2*^+/+^ and MMTV-Neu^+/+^/*Chn2*^−/−^. All genotypes were confirmed by PCR of mouse tail DNA at time of killing.

### Tumorigenesis analysis

MMTV-Neu^+/+^/*Chn2*^+/+^ and MMTV-Neu^+/+^/*Chn2*^−/−^ virgin females were monitored twice weekly for tumor development. Once detected, tumors were measured weekly with a digital caliper. Total tumor volume was calculated using the equation: volume = *a* × *b*^2^/2 where *a* is the longest dimension and *b* is the shortest. Animals were sacrificed 3 months after detection of the tumor unless the longest dimension of the largest tumor exceeded 2.5 cm, or if mice exhibited signs of illness. At necropsy, mammary tumors were collected, fixed in 4% buffered formaldehyde or snap-frozen in liquid nitrogen. Whole mounts of the remaining mammary glands from tumor-bearing mice were prepared as described below to quantify premalignant lesions. Tumor incidence was defined as the proportion of females that generated at least one mammary tumor during the experiment. Tumor growth was calculated by representing total tumor volume on a semilogaritmic scale and fitting by linear regression to calculate the slopes for each growth curve. Tumor burden was evaluated by assessing the total tumor volume per mouse.

For analysis of metastasis, lungs were collected, fixed in 4% buffered formaldehyde, and included in paraffin. The entire lungs were sectioned at 100 μm intervals, stained with H&E and scored for metastatic lesions.

### Mammary gland whole mounts analysis

Whole mounts of the mammary glands were prepared as previously described [47] and photographed using a stereomicroscope (Leica EZ4HD). Images were analyzed using ImageJ software. The mammary gland length was measured from the nipple area to the end of the longest duct. The number of terminal end buds (TEBs) was counted under the stereomicroscope at 8× magnification.

### Histological analysis and immunohistochemistry

Tumor specimens were paraffin-embedded, sectioned, and analyzed by H&E staining. Tumors were graded according to the modified Elston and Ellis grading system [48]. Immunohistochemistry was performed by independent personnel at the Histopathology units of either the CNIO (Madrid, Spain) or CIC (Salamanca, Spain). Proliferation and angiogenesis were monitored by immunohistochemical staining with antibodies against Ki67 (IR626, DAKO) and CD31 (IR610/IS610, DAKO), respectively. Apoptotic cells were detected using TUNEL labelling. E-cadherin staining was performed using anti E-cadherin (IR059) (DAKO). Images were acquired using a Nikon Eclipse 90i microscope.

### Western blot assay

Protein lysates from cells lines were prepared in a lysis buffer containing 50 mM Tris-HCl (pH 7.5), 150 mM NaCl, 1% Triton X-100, 0.2% DOC, 2mM EDTA, 1 mM Na_3_VO_4_, 1 mM DTT, 50 mM NaF, and a mixture of protease inhibitors (Cømplete, Roche Molecular Biochemicals). To prepare protein lysates from tumors, frozen tumor samples were homogenized using a polytron (Ultra Turrax) in the buffer lysis described above (1:10 w/v). Equivalent amounts of protein were resolved by SDS-PAGE and processed by immunoblotting analysis. The following primary antibodies were used: antibodies against E-cadherin (610181), β-catenin (D10A8), ZO-1 (D7D12), vimentin (D21H3), p-ERK (9101), ERK (9102), p-AKT (Ser473) (4060), AKT (9272) and p-p38 (Thr180/Tyr182) (9211) (Cell Signaling); cyclin D1 (sc-717) and p38 (sc-535) (Santa Cruz); Actin (A4700) and β2-chimaerin (HPA018989) (Sigma) and GFP (MMs-118P) (Covance). Immunoblot–derived signals were quantified using Quantity One-1D.

### Rac activation assay

Rac-GTP levels in cell lines and tumors were assessed by pull-down assay with a GST fusion protein containing the Rac1 binding domain of PAK1 (GST-PBD) as described previously [14]. Briefly, MCF7 cell lines were seeded in six well plates, serum starved for 48h and then stimulated with EGF (100 ng/ml) for 5 min or HRG (10 ng/ml) for 10 min. After stimulation cells were lysed in 400 μl of lysis buffer containing 20 mM Tris-HCl, pH 7.5, 150 mM NaCl, 5 mM MgCl_2_, 0.5% Igepal, 5 mM β-glycerophosphate, 1 mM DTT, 1 mM Na_3_VO_4_, 50 mM NaF, protease inhibitors (Cømplete, Roche Diagnostics), and 10 μg/sample of GST-PBD. Tumors were homogenized in the same lysis buffer (1:10 w/v) without GST-PBD, precleared by centrifugation at 14,000 rpm for 10 min at 4°C and then, 10 μg of GST-PBD was added to 1 ml of supernatant. Tumor and cell lysates were incubated with glutathione-Sepharose beads (GE Healthcare) for 1 h at 4°C. After incubation, samples were extensively washed, boiled in SDS-PAGE sample buffer and separated by electrophoresis. Bound Rac1 proteins were detected by immunoblotting using anti-Rac antibody (610651, BD transduction laboratories). Rac-GTP levels were quantitated using the Quantity One 1D image analysis software (Bio-Rad).

### Clinical dataset analysis

Gene expression analysis from GEO and TCGA databases were performed at the Bioinformatics Service at the University of Salamanca (Spain). *CHN2* and *CDH1* gene expression were retrieved from GEO databases containing normalized microarray data from normal breast and untreated Her2+ breast cancer samples (datasets GSE29431 and GSE65216). Differences in expression between normal breast and Her2+ samples were assessed by Student's t test followed by the Benjamini-Hochberg correction. The correlation between *CHN2* and *CDH1* expression in Her2+ patients was assessed using Pearson's correlation test. The SurvExpress tool was used to interrogate the prognostic value of *CHN2* mRNA expression in TCGA database [49]. The *P* value derived from a Student's t test analysis. The effect of *CHN2* expression on relapse-free survival and metastasis-free survival in unstratified breast cancer patients was evaluated using the online tool Kaplan-Meier Plotter and the log-rank test based on the updated 2014 version [41]. In all studies the probe set for *CHN2* was 213385_at, and for *CDH1* 201130_s_at.

### Statistical analyses

Tumor latency in Neu/β2WT and Neu/β2KO mice was compared using the Kaplan–Meier estimator and the log-rank test. Fisher's exact test was used to evaluate differences in grade distribution of tumors. Other analyses were performed with unpaired two-tailed Student's t-test, Mann-Whitney *U*-test or one way ANOVA.

## SUPPLEMENTARY FIGURES


