# Supplementary material for: A new role of the Rac-GAP β2-chimaerin in cell adhesion reveals opposite functions in breast cancer initiation and tumor progression
Source: Oncotarget. 2016 Apr 5;7(19):28301–19. doi: 10.18632/oncotarget.8597 (PMC5053728; doi:10.18632/oncotarget.8597)
Supplement: Supplementary file 1 [file oncotarget-07-28301-s001.pdf]

SUPPLEMENTARY FIGURES

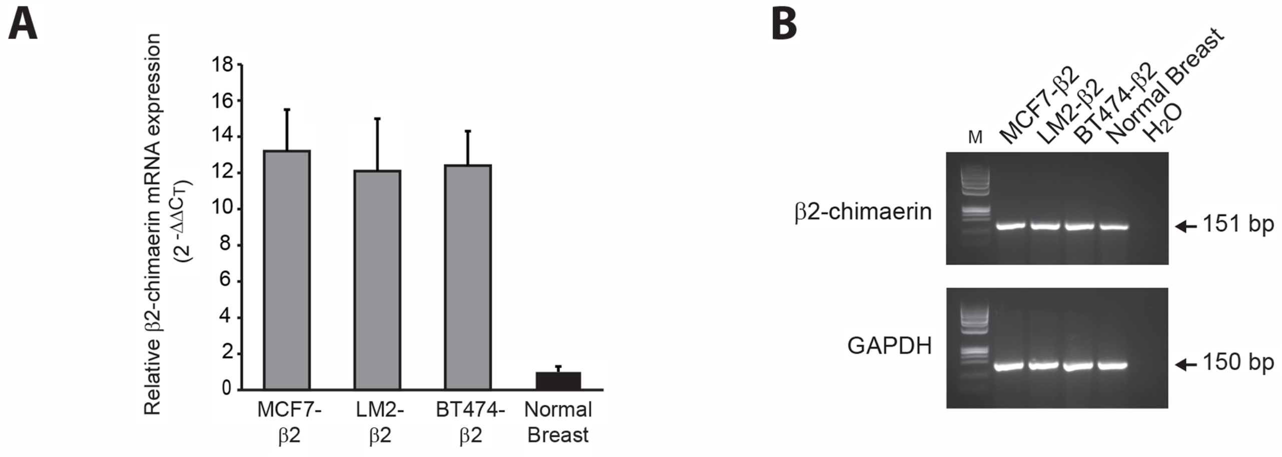

Supplementary Figure S1: Analysis of β2-chimaerin expression in MCF7-β2, LM2-β2 and BT474-β2 cell lines. A. Quantitative-RT-PCR analysis of β2-chimaerin expression in the indicated human breast cancer stable cell lines and in normal human breast tissue. The expression of GAPDHwas used for normalization. Relative β2-chimaerin expression levels in the cells lines compared to normal tissue were calculated using the $2^{-\Delta\Delta CT}$ method. The quantitative-RT-PCR was performed in triplicate. **B.** Electrophoretic analysis of the RT-PCR products. M: ΦX174 DNA/HaeIII marker.

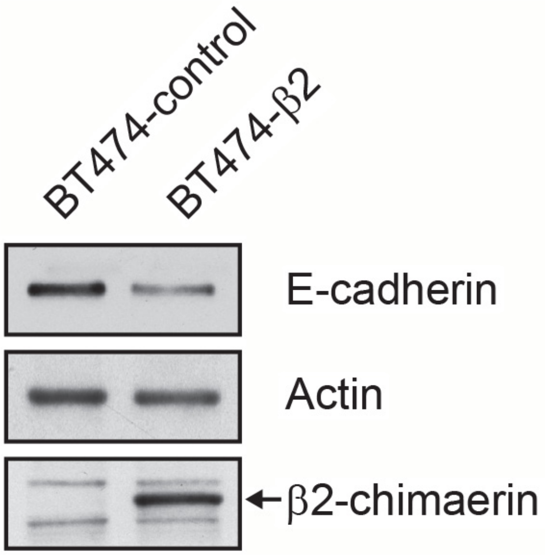

Supplementary Figure S2: β2-chimaerin decreases E-cadherin protein levels in breast cancer epithelial cells that overexpress the ErbB2 receptor. Cell lysates from BT474-control and BT474-β2 cells were analysed for expression of E-cadherin. Expression of actin was used as a loading control. Expression of β2-chimaerin-EGFP is shown in the lower panels.

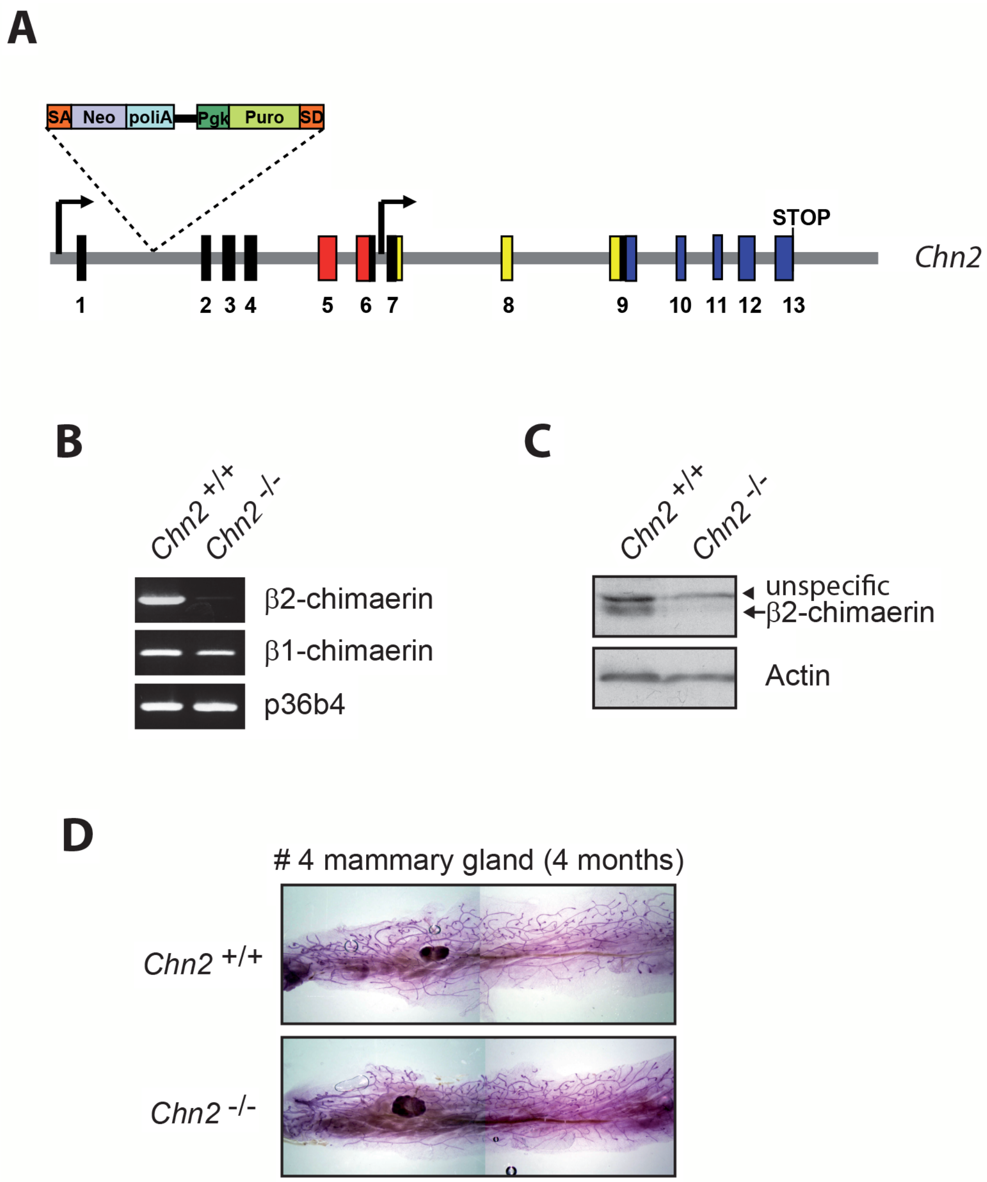

Supplementary Figure S3: Characterization of β2-chimaerin Gene Trap Mice. A. Schematic representation of the insertion of the gene trap for targeting β2-chimaerin. The *Chn2* gene contains two promoters (arrows) that drive the transcription of the β1- and β2-chimaerin isoforms. The gene trap was inserted upstream of the β1-chimaerin promoter. Exons coding for the SH2 domain are represented in red, for the C1 domain in yellow and for the GAP domain in blue. **B.** RT-PCR for β1- and β2-chimaerin isoforms from wild type (*Chn2*$^{+/+}$) and knockout (*Chn*$^{2-/-}$) mice showing the specific elimination of the β2-chimaerin mRNA in the *Chn*$^{2-/-}$ mice. **C.** Western blots analysis of β2-chimaerin expression in mammary gland lysates from adult *Chn2*$^{+/+}$ and *Chn*$^{2-/-}$ females. **D.** Whole mounts of inguinal mammary glands stained with alum carmine from 4 month-old virgin females of the indicated genotypes (8 x magnification).

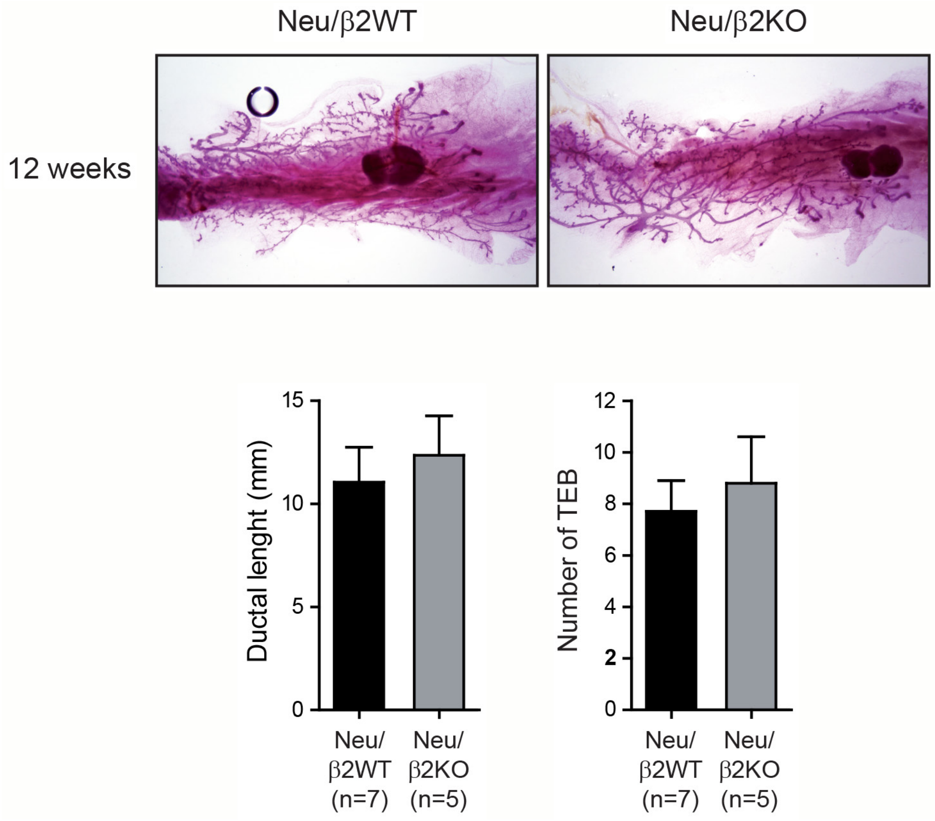

Supplementary Figure S4: β2-chimaerin deficiency in MMTV-Neu mice does not affect mammary gland architecture. Images show representative whole mounts of inguinal mammary glands from 12 week-old Neu/β2WT and Neu/β2KO females stained with alum carmine (8 x). Quantification of the ductal length and the number of tubular end buds (TEB) are shown in the histograms ($P = 0.43$ and $P = 0.68$ respectively by Mann Whitney U-test). Bars are means ± s.e.m. of 5-7 mice per genotype.
